# Structurally Orientated Rheological and Gut Microbiota Fermentation Property of Mannans Polysaccharides and Oligosaccharides

**DOI:** 10.3390/foods12214002

**Published:** 2023-11-01

**Authors:** Jing Wang, Sheng Ke, Padraig Strappe, Ming Ning, Zhongkai Zhou

**Affiliations:** 1 Key Laboratory for Processing and Quality Safety Control of Characteristic Agricultural Products, The Ministry of Agriculture and Rural Affairs, Shihezi University, Shihezi 832003, China; wangjing_996@126.com (J.W.); nming@shzu.edu.cn (M.N.); 2 College of Food Science and Engineering, Tianjin University of Science and Technology, Tianjin 300457, China; ks2929051598@163.com; 3 Curtin Health Innovation Research Institute (CHIRI), Curtin Medical School, Curtin University, Bentley, WA 6102, Australia; padraig.strappe@curtin.edu.au; 4 Gulbali Institute-Agriculture Water Environment, Charles Sturt University, Wagga, NSW 2678, Australia

**Keywords:** mannan polysaccharides, oligosaccharides, short-chain fatty acids, rheological property, gut microbiota, prebiotic function

## Abstract

Three mannan polysaccharides and their oligosaccharides were investigated in terms of physicochemical characteristics and effects on gut microbiota. Oligosaccharides from guar gum had the fastest fermentation kinetics for SCFAs generation at the initial stage, while the locust bean of both polymers and oligosaccharides demonstrated the lowest SCFAs through the whole fermentation process. In contrast, konjac gum steadily increased SCFAs and reached its maximum level at 24 h fermentation, indicating its fermentation character may be associated with its rheological properties. Compared to their corresponding polysaccharides, all the oligosaccharides demonstrated a faster fermentation kinetics, followed by an enriched abundance of propionate-producing bacterial *Prevotella* and a decreased abundance of *Megamonas* and *Collinsella*. Meanwhile, oligosaccharides reduced the *Firmicutes*/*Bacteroidota* ratio as well as the abundance of *Bacteroidetes* and *Escherichia-Shigella*. The fermentation of konjac substrate significantly promoted the abundance of butyrate-producing bacterial *Faecalibacterium*. In contrast, although the fermentation of locust bean and guar gum substrates benefited *Bifidobacterium* abundance due to their similar structure and monosaccharides composition, the fermentation of locust bean gum led to greater *Bifidobacterium* than the others, which may be associated with its higher mannose composition in the molecules. Interestingly, the partial hydrolysis of the three polysaccharides slightly reduced their prebiotic function.

## 1. Introduction

Locust bean gum, guar gum and konjac gum all belong to mannans polysaccharides, and are widely applied in the food industry as hydrocolloids [1]. Among them, konjac gum is a typical glucomannan, while locust bean gum and guar gum are galactomannans. As shown in Figure S1, the backbone of konjac gum is composed of D-mannose and D-glucose linked by β-D-1,4 bonds, and the side chains are glucose and acetyl groups. In contrast, guar gum and locust bean gum are homotypic polysaccharides, in which their main chains are formed by mannose through β-D-1,4 bonds, and their side chains consist of α-D-1,6-linked galactose. Structurally, guar gum contains more branched chains than locust bean gum [2,3,4]. The main chain of the mannan polysaccharides can be degraded into oligosaccharides by endo-β-mannanase via the breakage of the β-1,4 glycosidic bond, and a further degradation requires the synergistic action from β-mannosidase and β-glucosidase, as well as α-galactosidase and deacetylase for degrading the branched chains [1] (Figure S1). Given that humans lack the enzymes for hydrolyzing the β-1,4 glycosidic bonds [2], the mannan polysaccharides and their corresponding oligosaccharides can escape the digestive tract, and then reach the colon to be fermented by gut microbiota.

Indigestive carbohydrates, including dietary resistant starch, non-starch polysaccharides and oligosaccharides, are the major substrate to be utilized by gut microbiota. The consumption of these carbohydrates could induce changes in the composition of the gut microbiota, followed by changes in the production of short-chain fatty acids (SCFAs), such as acetic acid, propionic acid and butyric acid [5]. However, different carbohydrates demonstrated various impacts on both microbiota and the metabolites because of their corresponding structural diversity.

Although the physicochemical property of these mannose-containing polysaccharides has been well documented in term of their rheological property and gelling functionality, etc., an understanding of the interaction of these polysaccharides with gut microbiota on a molecular level is still small. In particular, following the wide application of their corresponding oligosaccharides in healthy foods as the prebiotics, investigations into differences in the gut microbiota fermentation properties between the polysaccharides and their corresponding hydrolysates (oligosaccharides) are in short supply too. Therefore, in this study, the changes in the physicochemical properties of the mannose-containing polysaccharides (konjac gum, locust bean gum and guar gum) before and after hydrolysis via *Bacillus amyloliquefaciens* secreted endo-β-mannanase were investigated. More importantly, the property of their gut microbiota and the major metabolites (SCFAs) following the fermentation of these three polysaccharides and their corresponding oligosaccharides was explored to reveal their molecularly manipulated mechanism. This study may highlight the importance of the individual application for either polysaccharides or their corresponding oligosaccharides.

## 2. Materials and Methods

### 2.1. Materials Subsection

Guar gum (G1) and locust bean gum (L1) were purchased from the Henan Fenglu Food Co., Ltd. (Zhengzhou, China), and konjac gum (K1) was purchased from the Hubei Johnson Konjac Gum Co., Ltd. (Wuhan, China). The SCFAs standards, acetic acid, propionic acid, butyric acid, and isobutyric acid were obtained from the Shanghai Aladdin Biochemical Technology Co., Ltd. (Shanghai, China). Dextrose T series standards (T-3, T-10, T-40, T-70 and T-500) were purchased from the Solarbio Co., Ltd. (Beijing, China). All other reagents were of analytical grade.

### 2.2. Preparation of Beta-Mannanases Solution

Using *Bacillus amyloliquefaciens* as the starting strain, the enzyme-producing medium included: glucose, 40 g/L; yeast powder, 9 g/L; ammonium sulfate, 0.8 g/L; magnesium sulfate, 0.1 g/L; dipotassium hydrogen phosphate, 2 g/L. After 80 h of fermentation (30 °C, 200 rpm), the enzyme solution was collected and its activity was determined by the DNS method [6] at 90 U/mL for subsequent oligosaccharide preparation.

### 2.3. Preparation of Mannan Oligosaccharides

Each mannan polysaccharide sample of 1 g was added into a 250 mL conical flask, followed by the addition of 100 mL of distilled water and enzyme solution (90 U). The hydrolysis was performed at 50 °C, at 200 rpm for 15 h, followed by inactivation in a boiling water bath for 10 min, and the supernatant was collected by centrifugation at 4000 rpm for 30 min (4 °C). The mannan oligosaccharides in the supernatant were prepared via spray drying.

### 2.4. Scanning Electron Microscopy (SEM) of the Samples

The morphology of all polysaccharides and oligosaccharides samples was observed using a scanning electron microscope (SU1510, Hitachi Ltd., Hitachi, Japan), and the images were recorded at an accelerating voltage of 5 KV with a magnification of either 2000×, respectively.

### 2.5. Particle Size Distribution of the Samples

The particle sizes of the three mannan polysaccharides and three corresponding oligosaccharides was determined with a laser particle size analyzer (Bettersize 2600, Baxter Instruments, Dandong, China). The samples were configured into a homogeneous suspension by adding drops to the cuvette, and the samples were then dispersed at a pump speed of 1600 rpm.

### 2.6. Molecular Weight (Mw) Analysis

The molecular weights (Mw) of polysaccharides and oligosaccharides were determined according to a previously reported method [7] with slight modifications. Briefly, a high-performance liquid chromatography (Agilent 1260, Agilent Technologies, Inc., Santa Clara, CA, USA) coupled with a TSKGEL column (Tosoh Co., Ltd., Tokyo, Japan) series G-4000 PWXL (7.8 × 300 mm) was used for the analysis. The operation conditions included the flow rate, column temperature and injection volume of the samples to be 0.6 mL/min, 30 ± 0.1 °C and 20 μL, respectively. The molecular weights of the polysaccharide and oligosaccharide were evaluated using the standard curves established from the T series dextran (T-3, T-10, T-40, T-70 and T-500).

### 2.7. Rheological Properties of Samples

The rheological properties of the samples were determined using a rheometer (HAAKE MARS 60, Thermo Fisher Scientific Inc., Germany) at 25 °C for viscoelasticity and apparent viscosity. The diameter of the test plate was 60 mm and the gap was set at 1 mm to measure the modulus of elasticity (G′) and viscosity modulus (G″) of the samples. Prior to the frequency scan test, the test sample was subjected to a strain scan test and its linear viscoelastic zone was determined. After determining its strain at 10%, the frequency scan test was performed in the range of 0.1–10 Hz. The apparent viscosity was determined with the test plate, and the variation of the shear rate was from 0–1000 s^−1^. Following the performance, the curves were fitted according to the Ostwald de Waele model, and the calculations were performed according to τ = Kɣ̇ⁿ.

### 2.8. Fourier Transform Infrared Spectroscopy (FTIR)

The FT-IR spectra of the samples were measured with a Fourier transform infrared spectrometer (FTIR Magna-IR IS50, Thermo Nicolet Co., USA). Briefly, the sample powder (1.0 mg) was mixed with KBr (150.0 mg), then ground and pressed into thin slices. The scanning range was 400–4000 cm^−1^ and the scanning frequency was 32 times.

### 2.9. In Vitro Gut Microbiota Fermentation

The in vitro fermentation of all the samples was performed according to a previous method [8]. Briefly, fresh feces were obtained from five volunteers (18.5 kg m^−2^ < BMI < 24 kg m^−2^, three males and two females, age between 20 and 25) with a normal diet, no digestive or chronic metabolic diseases, and no history of antibiotic treatment in the previous 3 months. Fecal samples were diluted threefold with a sterile phosphate buffered salt solution (PBS, pH 7.2–7.4). The base medium was prepared, which included L-cysteine hydrochloride (0.5 g/L), bile salts (0.5 g/L), Tween 80 (2 mL/L), vitamin K1 (10 µL/L), hemin (50 mg/L), peptone (2 g/L), yeast extract (2 g/L), NaHCO_3_ (2 g/L), CaCl_2_•6H_2_O (0.01 g/L), MgSO_4_•7H_2_O (0.01 g/L), NaCl (0.1 g/L), K_2_HPO_4_ (0.04 g/L), and KH_2_PO_4_ (0.04 g/L). The fecal suspensions were filtered through four layers of gauze and then mixed with the sterile base medium at 1:4 (initial pH of 6.78). Inserting 5 mL of fecal fermentation solution and 1% samples into each anaerobic tube, we then sealed the tube and transferred it to an anaerobic incubator for 6 h, 12 h, and 24 h incubation at 37 °C, respectively. After the fermentation, the samples were stored at −80 °C and subsequently subjected to 16S rDNA gene sequencing analysis. The samples were referred to as K1 (konjac mannan polysaccharides), K2 (konjac oligosaccharides), G1 (guar mannan polysaccharides), G2 (guar oligosaccharides), L1 (locust bean mannan polysaccharides), and L2 (locust bean oligosaccharides), respectively. Each experiment was performed in triplicate. Informed consent was obtained from all volunteers involved in this study, and the experimental procedure was approved by the university ethics committee.

### 2.10. Determination of SCFAs during the Gut Microbiota Fermentation Progress

After the incubation, the fecal fermentation solution was centrifuged at 10,000 rpm for 15 min, and then 500 μL of the supernatant was withdrawn and mixed with 10 μL of 5% (*v*/*v*) H_2_SO_4_ and shaken for 30 s, followed by the addition of 800 μL of ether. The mixture was shaken for 30 s and then centrifuged at 10,000 rpm for 20 min at 4 °C. The supernatant was collected and filtered (0.22 μm), and the SCFAs content was determined by gas chromatography (GC-2014, SHIMADZU Co., Ltd., Kyoto, Japan). The column temperature was maintained at 100 °C for 3 min, and then increased to 190 °C at 6 °C/min and maintained at 190 °C for 10 min. The gasification chamber temperature and the hydrogen ion flame detector temperature were set to be 230 °C and 275 °C, respectively. The capillary column used was Nukol™ 30 m × 0.53 mm × 0.5 μm film thickness (SUPELCO, USA).

### 2.11. 16S rDNA Gene Sequencing Analysis

The 16S rDNA gene sequencing was performed by the Shanghai Baiqu Biomedical Technology Co., Ltd. The microbial DNA was extracted from fecal samples, and the 16S rDNA V3-V4 region was amplified by PCR, and then sequenced with NovaSeq6000. Paired-end reads were assigned to samples based on their unique barcode and truncated by cutting off the barcode and primer sequence. Paired-end reads (raw tags) were merged using FLASH (V1.2.7). Quality filtering on the raw tags was performed under specific filtering conditions to obtain high-quality clean tags according to the QIIME (V1.9.1) quality controlled process. The tags were compared with the reference database (Silva database) using UCHIME Algorithm to detect chimera sequences, and the chimera sequences were then removed. Sequences analysis was performed by Uparse software (Uparse v7.0.1001).

### 2.12. Statistical Analysis

All the data were expressed as mean ± standard error. The experimental results were analyzed using SPSS 19.0 software with an analysis of variance (ANOVA) for the significance of the difference (*p* < 0.05). Origin 9.0 soft and ChemDraw 20.0 software were used for graphing. Heatmaps and correlation heatmaps were graphed by Omicshare (https://www.omicshare.com/ (accessed on 21 May 2023)).

## 3. Results and Discussion

### 3.1. Microstructure, Molecular Weight and Particle Size Distribution of Mannan Polysaccharides and Oligosaccharides

**Figure 1:**
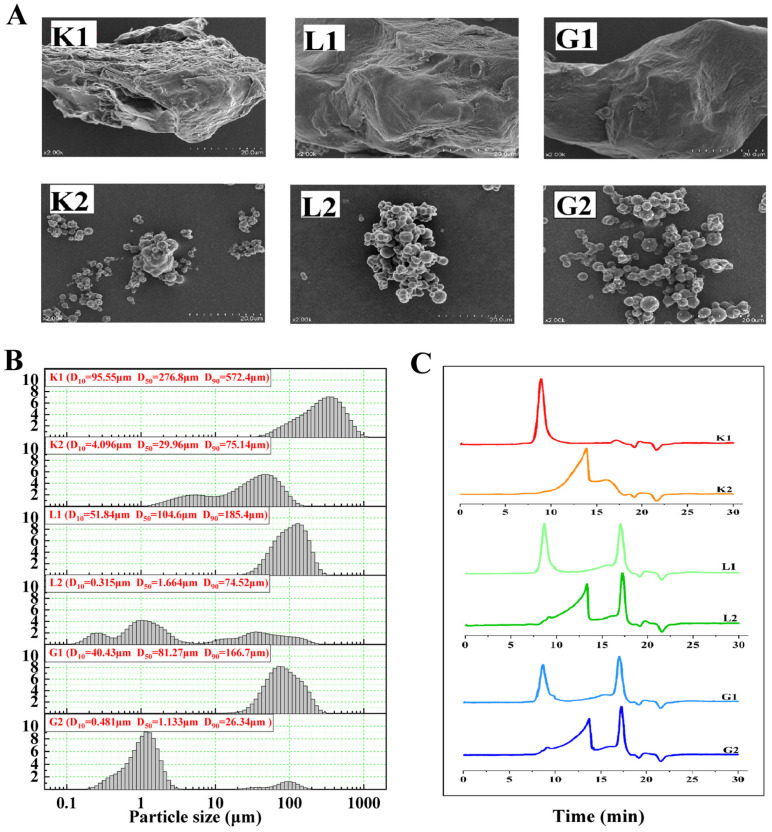
Microstructure of mannan polysaccharides and oligosaccharides. (**A**) particle size distribution; (**B**) molecular weights; (**C**) particles morphology. K1: konjac mannan polysaccharides; K2: konjac oligosaccharides; G1: guar mannan polysaccharides; G2: guar oligosaccharides; L1: locust bean mannan polysaccharides; L2: locust bean oligosaccharides. D50: The particle size corresponding to a sample with a cumulative percent size distribution of 50%.

Considering that the morphological property and particle size distribution of the mannan polysaccharide and oligosaccharide samples could influence their interaction with the gut microbiota, their corresponding particle microstructure was therefore also determined as displayed in Figure 1. It can be seen that all the polysaccharides particles (K1, G1 and L1) showed an irregular shape, in which sample K1 had a rougher surface and a larger particle diameter compared to the other two polysaccharides. Sample L1 and G1 had a smoother surface and a similar particle size distribution between them. Interestingly, all the oligosaccharides (K2, G2 and L2) appeared as round spheres with a significantly smaller granule diameter compared to their corresponding polysaccharides particles. The difference in the impact of the hydrolysis of the polysaccharides on particle size distribution was noted to be shifted from a single peak (polysacchrides) to multiple peaks (oligosaccharides) (Figure 1B). The hydrolysis led to the D50 of K1, G1 and L1 being reduced by 89.18%, 98.41% and 98.61%, respectively, of which the D50 of L1 experienced the largest decrease. Data in Figure 1C indicated the HPLC chromatography of polysaccharides and oligosaccharides, and the molecular weight (Mw) changed from 3788 to 56 kDa for the hydrolysis of konjac, from 4514 to 90 kDa for the hydrolysis of the locust bean, and from 4439 to 67 kDa for the hydrolysis of the guar gum, where konjac oligosaccharides had the lowest molecular weight, which may be associated with its largest particle size.

### 3.2. Rheological Properties of Mannans Polysaccharides and Oligosaccharides

**Figure 2:**
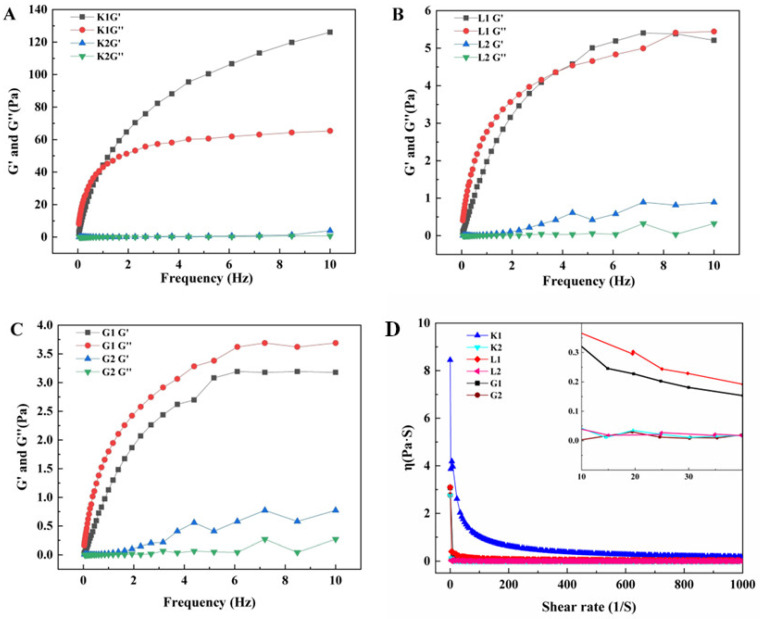

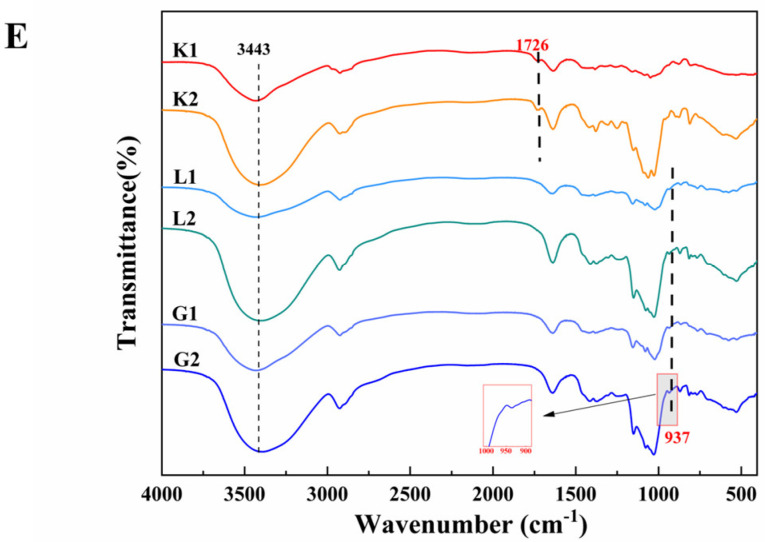
Rheological and infrared properties of polysaccharides and oligosaccharides. (**A**–**C**) rheological properties; (**D**) apparent viscosity; (**E**) FT–IR spectra. K1: konjac mannan polysaccharides; K2: konjac oligosaccharides; G1: guar mannan polysaccharides; G2: guar oligosaccharides; L1: locust bean mannan polysaccharides; L2: locust bean oligosaccharides.

Data in Figure 2A–D indicate the difference in the dynamic rheological properties of the three mannan polysaccharides and their corresponding oligosaccharides. This study revealed that sample K1 had the highest G′ and G″ compared to other samples in terms of G1 and L1. Furthermore, the G′ value was higher than G″ for K1 (Figure 2A), indicating that it was a gel state with a three-dimensional network structure, while G″ > G′ for G1 (Figure 2C), suggesting a liquid state. In contrast, the crossover of G′ and G″ was noted for L1 (Figure 2B), which may imply the occurrence of the transition of the sample from the gel to the liquid state during the shear. Furthermore, K1 exhibited larger G′ and G″ values, which may be attributed to the unique acetyl group in the structure of the K1 molecule, because the acetyl groups could enhance the gelling properties via the inhibition of the formation of intramolecular hydrogen bonds, accompanied by an increase in the hydrogen bonds with water molecules and a reduced hydrophobic interaction [9]. The G′ and G″ values for all the oligosaccharides (K2, G2 and L2) experienced a significant decrease, and the values were very close to each other, indicating that the hydrolysis destroyed the three-dimensional network structure inside the gel.

The apparent viscosity of the mannan polysaccharides and oligosaccharides is shown in Figure 2D. The apparent viscosity of all the samples was decreased followed by the increased shear rate, exhibiting a typical shear thinning behavior. The viscosity of the K1 solution was greater than that of G1 and L1. The viscosity of the three oligosaccharides was much less than that of their corresponding polymers. Furthermore, the shear rate of the polysaccharides was greater than the recovery rate of the intermolecular hydrogen bonds, resulting in an alignment of the polysaccharide molecules along the direction of the shear stress and a decrease in structural cross-linking.

### 3.3. FT–IR Spectra of Polysaccharides and Oligosaccharides

Results in Figure 2E demonstrate the IR spectra of the mannan polysaccharides and their corresponding oligosaccharides, in which the absorption peak at 3443 cm^−1^ belongs to the −OH stretching vibration, and the hydrolysis enhanced the peak intensity of −OH, which was attributed to the exposure of more −OH after the breakage of the glycosidic bond. Importantly, the absorption peak at 937 cm^−1^ attributed to D–galactose was a characteristic absorption for distinguishing the substrate G and L from the substrate K. Furthermore, the absorption of K1 at 1726 cm^−1^ was assigned to the carbonyl groups (acetyl groups), and it was still maintained for K2 following the hydrolysis, indicating that the hydrolysis only changed their molecular weight rather than their composition.

### 3.4. Production of SCFAs following the Gut Microbiota Fermentation

**Figure 3:**
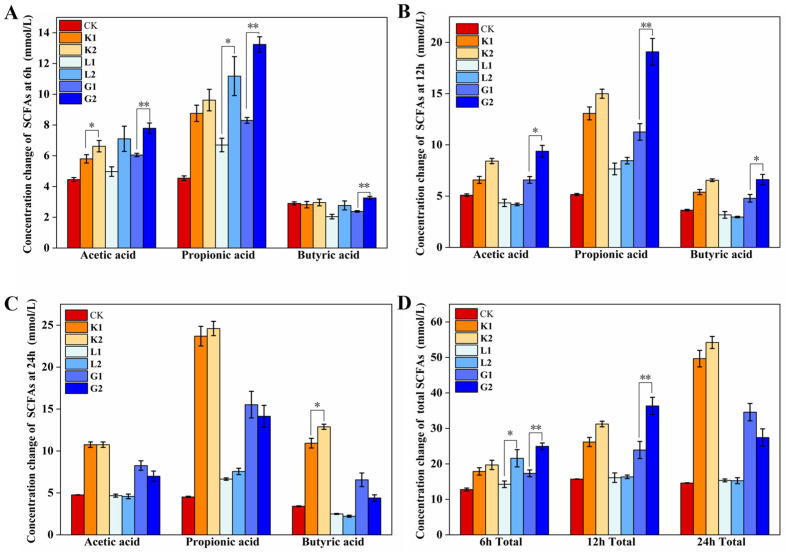
Changes in individual and total SCFAs following the gut microbiota fermentation at different intervals. (**A**) for 6 h; (**B**) for 12 h; (**C**) for 24 h; (**D**) total SCFAs for 6, 12, 24 h. K1: konjac mannan polysaccharides; K2: konjac oligosaccharides; substrate K (K1 and K2); G1: guar mannan polysaccharides; G2: guar oligosaccharides; substrate G (G1 and G2); L1: locust bean mannan polysaccharides; L2: locust bean oligosaccharides; substrate L (L1 and L2); CK: blank samples without either polysaccharides or oligosaccharides added; * indicates *p* < 0.05, ** indicates *p* < 0.01, respectively.

As revealed above, although the enzymatic hydrolysis of the polysaccharides altered their molecular weight rather than their composition, leading to a different rheological property between polymers and oligosaccharides, the changes in the rheological property may manipulate their interaction with the gut microbiota to subsequently influence the microbiota profile and the metabolites. As shown in Figure 3, at a fermentation time of 6 h (Figure 3A), the total short-chain fatty acid concentration produced by the three types of oligosaccharides was 19.21 ± 1.28 (K2), 24.29 ± 0.93 (G2) and 21.05 ± 2.39 mmoL/L (L2), respectively; these were significantly higher than their corresponding polysaccharides (17.38 ± 1.01 for K1, 16.73 ± 0.35 for G1, and 13.71 ± 0.90 mmoL/L for L1, respectively), except for the sample K2 vs. sample K1, indicating that the oligosaccharides with a smaller molecular size were more preferentially utilized by the microbiota compared to their corresponding polysaccharides. More interestingly, the release of SCFAs in G1 continued as the fermentation time increased. Substrate G2 had the highest release of SCFAs at 12 h and its content then decreased at 24 h (Figure 3B,C), which may be related to a re-utilization of the SCFAs caused by microbiota for its growth [10]. Importantly, substrate K (K1 and K2) generated the highest SCFAs among the groups, which may be partially related to the release of acetyl groups to contribute to the acetate production from its molecules [2]. Meanwhile, the slow fermentation property of the substrate K, which could endure a 24 h fermentation process, may be another factor contributing to its higher SCFAs generation, and its fermentation kinetics may be associated with its high G′ and G″ as evidenced by the rheological property in this study (Figure 2A–C), which may in turn be associated with the existence of the acetyl group in its molecules compared to the other two polysaccharides. This study also revealed that the existence of the substrate K (K1 and K2) maintained a higher butyrate and propionate even following a 24 h fermentation (Figure 3B,C). These differences may be associated with their gut microbiota characteristics.

### 3.5. Changes in the Gut Microbiota following the Fermentation

#### 3.5.1. Difference in Alpha and Beta Diversity following the Fermentation

The differences in the indices of *alpha*-diversity of the gut microbiota following the mannans polysaccharides and oligosaccharides fermentation are shown in Table S1. The results showed that the Shannon and Simpson indices of the polysaccharides and oligosaccharides samples were lower than the control, indicating a decrease in community diversity, which may be due to the generation of the SCFAs to suppress the growth of some bacterial varieties; a similar phenomenon was also observed previously for the study of *Pleurotus eryngii* polysaccharides [11]. Among them, Shannon and Simpson indices were significantly higher (*p* < 0.05) in G2 and L2 than their corresponding polysaccharides, and similarly, ACE and Chao1 indices were also consistently higher in both G2 and L2 than their corresponding polysaccharides, suggesting that the molecular weight of the carbohydrates provides a facilitating effect on improving intestinal flora diversity and richness. In contrast, there was no significant difference in those indices for the gut microbiota following the fermentation between K1 and K2.

**Figure 4:**
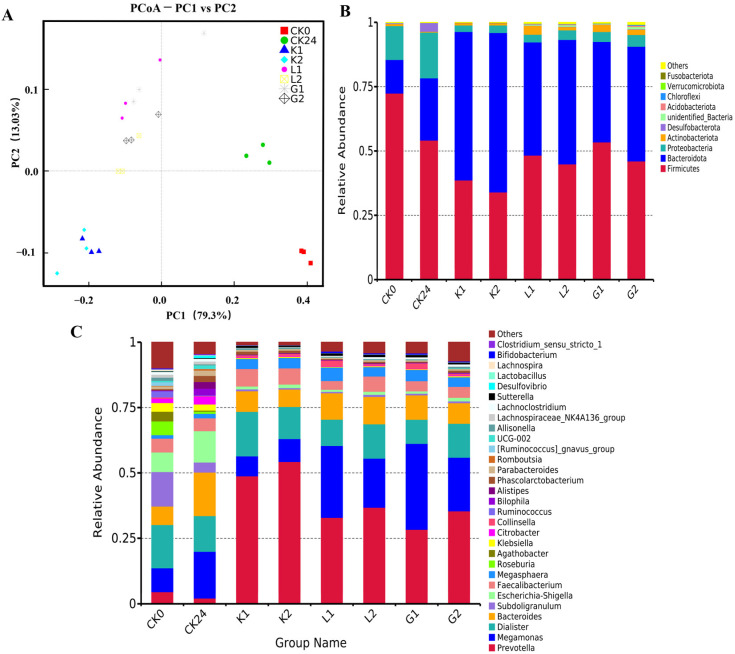
Comparison of the intestinal flora composition of mannan polysaccharides and oligosaccharides samples following a 24 h fermentation. (**A**) beta diversity indices; (**B**) bacterial abundance on phylum level; (**C**) bacterial abundance on genus level. CK0: blank control at 0 h fermentation; CK24: blank control at 24 h fermentation; K1: konjac polysaccharides; K2: konjac oligosaccharides; substrate K (K1 and K2); G1: guar polysaccharides; G2: guar oligosaccharides; substrate G (G1 and G2); L1: locust bean polysaccharides; L2: locust bean oligosaccharides; substrate L (L1 and L2).

Furthermore, the difference in the indices of *beta*–diversity of the gut microbiota following the mannans polysaccharides and oligosaccharides fermentation, respectively, is shown in Figure 4A. The cumulative variance contribution of the two principal components was 92.33%, with PC1 and PC2 accounting for 79.3% and 13.03% of the total variance, respectively. Compared to the blank control (CK0, CK24), a significant separation was achieved among the substrate K (K1 and K2), substrate L (L1 and L2) and substrate G (G1 and G2), indicating that the fermentation of mannan polysaccharides and oligosaccharides promoted changes in the composition of the intestinal microbiota. Samples L2 and G2 were clearly separated from samples L1 and G1, suggesting that the hydrolysis of polysaccharides L1 and G1 generated a greater impact on their corresponding fermentation characteristics. In contrast, sample K2 was closer to K1, showing that enzymatic hydrolysis did not have a significant influence on the fermentation of the substrate K1.

#### 3.5.2. Changes in the Gut Microbiota on Phylum Levels following the Fermentation

Data in Figure 4B indicate the taxonomic changes in the intestinal bacteria on a phylum level following a 24 h fermentation of various substrates. Compared to the blank control, the fermentation of either polysaccharides or oligosaccharides increased the relative abundance of *Bacteroidota* and *Actinobacteriota* and decreased *Firmicutes* and *Proteobacteria* abundance. Compared to their corresponding mannan polysaccharides, the fermentation of all the oligosaccharides promoted the abundance of *Bacteroidota*, with the highest relative abundance in substrate K, followed by substrate L and the lowest in substrate G. More interestingly, among the polysaccharide groups, the fermentation of K1 and L1 significantly (*p* < 0.05) reduced the *Firmicutes*, but not for G1. In contrast, the fermentation of the oligosaccharides indicated that both K2 and G2 led to a remarkably decreased *Firmicutes*, but not for L2. Importantly, the addition of either mannans polysaccharides or oligosaccharides significantly (*p* < 0.05) reduced the ratio of the *Firmicutes* to *Bacteroidota* (F/B) compared to the control group. Among the polysaccharide samples, K1 had the lowest F/B value. However, no significant difference between G1 and L1 was noted, which may be due to the similarity of the monosaccharides in their molecules. Nevertheless, the fermentation of the oligosaccharides further reduced the F/B value compared to the corresponding polysaccharides, suggesting that the difference in the physiological functions could be achieved by controlling their molecular weights. Previous studies suggested that the significant increase in the *Bacteroidetes* may be attributed to the fact that the *Bacteroidetes* encodes more glycoside hydrolases, polysaccharide lyases and surface enzymes than the *Firmicutes*, while the *Firmicutes* has more polysaccharide transporters than *Bacteroidetes* [12,13]. Thus, *Bacteroidetes* could grow faster following the polysaccharides degradation, while the *Firmicutes* increases the utilization of oligosaccharides for growth by improving translocation efficiency. A cross-feeding between the *Bacteroidetes* and *Firmicutes* may additionally occur [14]. Nevertheless, the relative abundance of *Proteobacteria* was reduced in all samples, indicating the potential function of the fermentation of mannans polysaccharides and their corresponding oligosaccharides in improving microbial dysbiosis and preventing the pathological property of the gut [15].

#### 3.5.3. Changes in the Gut Microbiota on Genus Level following the Fermentation

**Figure 5:**
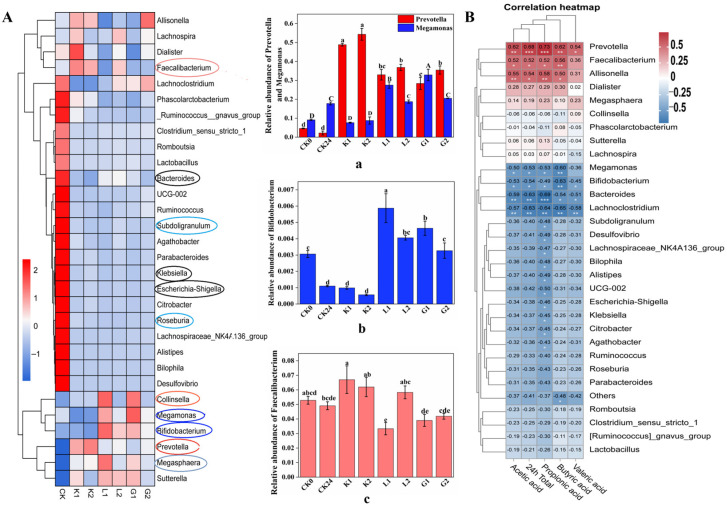
(**A**) Heat map analysis of the relative abundance of bacterial communities at the genus level; (**Aa**) *Faecalibacterium*; (**Ab**) *Bifidobacterium*; (**Ac**) *Prevotella* and *Megalomonas*; (**B**) Spearman correlation analysis between flora and SCFAs. CK0: blank control at 0 h fermentation; CK24: blank control at 24 h fermentation; K1: konjac polysaccharides; K2: konjac oligosaccharides; substrate K (K1 and K2); G1: guar polysaccharides; G2: guar oligosaccharides; substrate G (G1 and G2); L1: locust bean polysaccharides; L2: locust bean oligosaccharides; substrate L (L1 and L2); different lowercase letters indicate significant difference between samples (*p* < 0.05); (Pearson correlation) * *p* < 0.05, ** *p* < 0.01, *** *p* < 0.001; (**Aa**–**Ac**) different letter in each column indicates significant difference.

Data in Figure 4C indicate the changes in the relative abundance of the top 30 genera of each sample following 24 h fermentation. Meanwhile, a heat map analysis of the intestinal microbial community was also established (Figure 5A) for demonstrating the effects of the structure, monosaccharide composition and enzymatic digestion of the substrates on the intestinal flora. As shown in Figure 5(Ac), *Prevotella* and *Megamonas* were two important genera following the fermentation of both polysaccharides and oligosaccharides samples. Compared to the control group, the fermentation of either polysaccharides or oligosaccharides samples led to an enhanced *Prevotella* abundance, which was consistent with previous report [16]. Nevertheless, the fermentation of oligosaccharides further increased the abundance of *Prevotella* compared to the corresponding polysaccharides, with the most significant increase in the sample G2. Among them, the highest relative abundance was found in substrate K. That may be due to its containing multiple genes for encoding active enzymes to degrade the polymers [17,18]. Considering that *Prevotella* has been reported to play roles in improving glucose metabolism by increasing glycogen stores [19], this study further revealed that, compared to the polysaccharides, the fermentation of the oligosaccharides by the gut microbiota seemed to generate a specific physiological function.

*Megamonas* belong to the *Firmicutes*, which utilize D-mannose, lactose, and glucose [20]. In the present study, the abundance of the *Megamonas* increased significantly (*p* < 0.05) following the fermentation of substrate G1 and L1. In contrast, it decreased significantly (*p* < 0.05) following the fermentation of K1. A previous study also indicated that the presence of α-galactosidase in *Megamonas* could increase the amount of substrate availability during the fermentation for either substrate L or G. In contrast, *Megamonas* lacks β-glucosidase for breaking down the glucosidic bond in the substrate K molecules [20]. Therefore, compared to sample K, the structural characteristics of sample G and L in terms of a higher galactose content and the side chain may stimulate the abundance of *Megamonas*, followed by a faster generation of SCFAs at the initial fermentation stage (e.g., 6 h). In contrast, a lower abundance in the *Megamonas* following the fermentation of substrate K may be partially related to a lower breakage of the glucosidic bond in its molecules, accompanied by a slower fermentation kinetics as discussed above.

As shown in Figure 5(Aa), the *Faecalibacterium* has been reported to be incapable of utilizing mannose as a growth substrate, indicating that it cannot be the dominant genus for the three mannans and oligosaccharides. However, its ability to utilize glucose and acetic acid for growth may explain why it exhibits substrate K as the preferred one rather than G and L [21]. Furthermore, this study revealed that, among the oligosaccharides, fermentation of K2 led to the greatest abundance of *Faecalibacterium* (*p* < 0.05), followed by L2, but not in sample G2. More importantly, this is the first report to find that, as shown in Figure 5A, *Megasphaera* was selected to be significantly (*p* < 0.05) enriched following the fermentation of the six substrates, and the fermentation of the six substrates could strongly depress the growth of *Klebsiella*. Meanwhile, as shown in Figure 5(Ab), *Bifidobacterium* was also significantly (*p* < 0.05) enriched in L and G substrates, but not in K substrates following the fermentation, which may be due to their faster fermentation kinetics for samples L and G. *Roseburia* and *Subdoligranulum* (Figure 5A) were reported to be unable to utilize mannose, which may be the main reason for a reduced relative abundance of this genus [22,23]. Furthermore, *Bacteroides* have been reported to be associated with many gastrointestinal diseases and pathogens [24], while the lack in recognition and degradation of the complex polysaccharide enzymes and gene clusters may be the key contributors to its lower abundance following the fermentation. Interestingly, *Collinsella* was exhibited to be enriched in L1 and G1. In contrast, no significant enrichment was observed in their corresponding oligosaccharides L2 and G2 for showing a molecular chain length-dependent fermentation manner [25].

#### 3.5.4. Spearman Correlation Analysis between Flora and SCFAs

**Figure 6:**
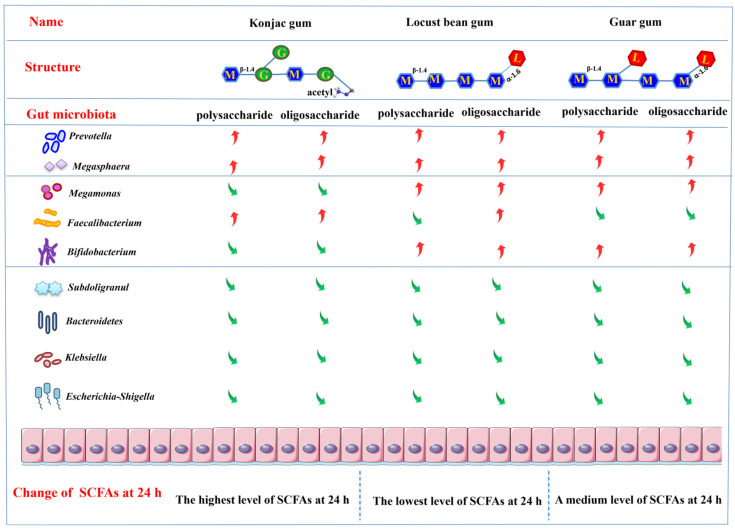
A proposed mechanism of the regulation on microbiota and metabolites following the fermentation of three mannose polysaccharides and oligosaccharides.

The correlation between the production of SCFAs and the growth of intestinal microorganisms during polysaccharide and oligosaccharide fermentation is established by Spearman correlation analysis as shown in Figure 5B. The results showed that the abundance of *Prevotella*, *Faecalibacterium* and *Allisonella* was significantly and positively correlated with the concentrations of acetic acid, propionic acid, butyric acid and total SCFA. In contrast, *Megamonas*, *Megasphaera*, *Bacteroides* and *Lachnoclostridium* showed opposite correlations for the content of almost all SCFAs. Among them, *Prevotella* (*p* < 0.001) had a very strong correlation with propionic acid, and *Faecalibacterium* (*p* < 0.01) demonstrated a stronger correlation with butyric acid and *Allisonella* (*p* < 0.01) showed a strong correlation with both acetic and butyric acid, indicating their importance for maintaining the gut environment in term of metabolites and microbiota profile (Figure 6).

## 4. Conclusions

This research investigated the difference in the SCFAs production and the gut microbiota profile following the fermentation with different structures of mannan polysaccharides (K1, L1 and G1) and oligosaccharides (K2, L2 and G2). The utilization of polysaccharides by microorganisms resulted in an increased production of SCFAs, with substrate K having a higher total SCFAs content than substrates L and G. In particular, K2 produced the highest total SCFAs of all groups during 24 h of fermentation. In terms of the relative abundance of the flora, bacteria with a complete catalytic mechanism for glycolysis become the dominant genus e.g., *Prevotella*, followed by genera with a partial degradation mechanism but one capable of utilizing multiple monosaccharides in the structure e.g., *Megamonas*. Then come bacteria that can utilize only one monosaccharide or can be selectively enriched by cross-feeding e.g., *Faecalibacterium*. On the other hand, the fermentation of all the carbohydrate substrates led to a reduced abundance of *Subdoligranulum* compared to the control sample. Based on the results, the association between the utilization of carbohydrates and the enrichment of the key bacteria is also proposed in the current study (Figure 6). In summary, the fermentation of the corresponding oligosaccharides mainly increased the relative abundance of the major genera, such as *Prevotella*, and decreased the abundance of some specific genera compared to polysaccharides, such as *Megamonas* and *Collinsella*. The difference in the molecular size and the monosaccharides composition of the mannans polysaccharides may play different roles in the improvement of the intestinal microenvironment.

## Data Availability

The data presented in this study are available on request from the corresponding author.

## Supplementary Materials

The following supporting information can be downloaded at: https://www.mdpi.com/article/10.3390/foods12214002/s1, Table S1: *Alpha*-diversity index of mannan polysaccharides and oligosaccharides following 24 h fermentation; Figure S1: Molecular structure characteristics and their corresponding degradation pattern of konjac gum, guar gum and locust bean gum.

## References

[1] Moreira L., Filho E. (2008). An overview of mannan structure and mannan-degrading enzyme systems. Appl. Microbiol. Biotechnol..

[2] Behera S.S., Ray R.C. (2016). Konjac glucomannan, a promising polysaccharide of *Amorphophallus konjac* K. Koch in health care. Int. J. Biol. Macromol..

[3] Hamdani A.M., Wani I.A. (2017). Guar and Locust bean gum: Composition, total phenolic content, antioxidant and antinutritional characterisation. Bioact. Carbohydr. Diet. Fibre.

[4] Katsuraya K., Okuyama K., Hatanaka K., Oshima R., Sato T., Matsuzaki K. (2003). Constitution of konjac glucomannan: Chemical analysis and 13C NMR spectroscopy. Carbohydr. Polym..

[5] La Rosa S.L., Leth M.L., Michalak L., Hansen M.E., Pudlo N.A., Glowacki R., Pereira G., Workman C.T., Arntzen M.Ø., Pope P.B. (2019). The human gut Firmicute *Roseburia intestinalis* is a primary degrader of dietary β-mannans. Nat. Commun..

[6] Du G., Qing Y., Wang H., Wang N., Yue T., Yuan Y. (2023). Effects of Tibetan kefir grain fermentation on the physicochemical properties, phenolics, enzyme activity, and antioxidant activity of *Lycium barbarum* (Goji berry) juice. Food Biosci..

[7] Liu Y., Duan X., Duan S., Li C., Hu B., Liu A., Wu Y., Wu H., Chen H., Wu W. (2020). Effects of in vitro digestion and fecal fermentation on the stability and metabolic behavior of polysaccharides from *Craterellus cornucopioides*. Food Funct..

[8] del Hierro J.N., Cueva C., Tamargo A., Núñez-Gómez E., Moreno-Arribas M.V., Reglero G.J., Martin D. (2019). In vitro colonic fermentation of saponin-rich extracts from quinoa, lentil, and fenugreek. Effect on sapogenins yield and human gut microbiota. J. Agric. Food Chem..

[9] Guo Q., Zhu X., Zhen W., Li Z., Kang J., Sun X., Wang S., Cui S.W. (2021). Rheological properties and stabilizing effects of high-temperature extracted flaxseed gum on oil/water emulsion systems. Food Hydrocoll..

[10] Carlson J.L., Erickson J.M., Hess J.M., Gould T.J., Slavin J.L. (2017). Prebiotic dietary fiber and gut health: Comparing the in vitro fermentations of beta-glucan, inulin and xylooligosaccharide. Nutrients.

[11] Ma G., Xu Q., Du H., Kimatu B.M., Su A., Yang W., Hu Q., Xiao H. (2022). Characterization of polysaccharide from *Pleurotus eryngii* during simulated gastrointestinal digestion and fermentation. Food Chem..

[12] Kaoutari A.E., Armougom F., Gordon J.I., Raoult D., Henrissat B. (2013). The abundance and variety of carbohydrate-active enzymes in the human gut microbiota. Nat. Rev. Microbiol..

[13] Cockburn D.W., Koropatkin N.M. (2016). Polysaccharide degradation by the intestinal microbiota and its influence on human health and disease. J. Mol. Biol..

[14] Hamilton A.L., Kamm M.A., Ng S.C., Morrison M. (2018). *Proteus* spp. as putative gastrointestinal pathogens. Clin. Microbiol. Rev..

[15] Dominguez-Bello M.G., De Jesus-Laboy K.M., Shen N., Cox L.M., Amir A., Gonzalez A., Bokulich N.A., Song S.J., Hoashi M., Rivera-Vinas J.I. (2016). Partial restoration of the microbiota of cesarean-born infants via vaginal microbial transfer. Nat. Med..

[16] Kovatcheva-Datchary P., Nilsson A., Akrami R., Lee Y.S., De Vadder F., Arora T., Hallen A., Martens E., Björck I., Bäckhed F. (2015). Dietary fiber-induced improvement in glucose metabolism is associated with increased abundance of *Prevotella*. Cell Metab..

[17] De Filippo C., Cavalieri D., Di Paola M., Ramazzotti M., Poullet J.B., Massart S., Collini S., Pieraccini G., Lionetti P. (2010). Impact of diet in shaping gut microbiota revealed by a comparative study in children from Europe and rural Africa. Proc. Natl. Acad. Sci. USA.

[18] Dodd D., Mackie R.I., Cann I.K. (2011). Xylan degradation, a metabolic property shared by rumen and human colonic Bacteroidetes. Mol. Microbiol..

[19] Takahashi N., Yamada T. (2000). Glucose metabolism by *Prevotella intermedia* and *Prevotella nigrescens*. Oral Microbiol. Immun..

[20] Sakon H., Nagai F., Morotomi M., Tanaka R. (2008). *Sutterella parvirubra* sp. nov. and *Megamonas funiformis* sp. nov. isolated from human faeces. Int. J. Syst. Evol. Microbiol..

[21] Heinken A., Khan M.T., Paglia G., Rodionov D.A., Harmsen H.J., Thiele I. (2014). Functional metabolic map of *Faecalibacterium prausnitzii*, a beneficial human gut microbe. J. Bacteriol..

[22] Hillman E.T., Kozik A.J., Hooker C.A., Burnett J.L., Heo Y., Kiesel V.A., Nevins C.J., Oshiro J.M., Robins M.M., Thakkar R.D. (2020). Comparative genomics of the genus *Roseburia* reveals divergent biosynthetic pathways that may influence colonic competition among species. Microb. Genomics.

[23] Holmstrøm K., Collins M.D., Møller T., Falsen E., Lawson P.A. (2004). *Subdoligranulum variabile* gen. nov. sp. nov. from human feces. Anaerobe.

[24] Wexler H.M. (2007). Bacteroides: The good, the bad, and the nitty-gritty. Clin. Microbiol. Rev..

[25] Wongkuna S., Ghimire S., Chankhamhaengdecha S., Janvilisri T., Scaria J. (2021). Description of *Collinsella avium* sp. nov. a new member of the *Collinsella* genus isolated from the ceacum of feral chicken. New Microbes New Infect..

