# Supplementary material for: Structurally Orientated Rheological and Gut Microbiota Fermentation Property of Mannans Polysaccharides and Oligosaccharides
Source: Foods. 2023 Nov 1;12(21):4002. doi: 10.3390/foods12214002 (PMC10649220; doi:10.3390/foods12214002)

Table S1 *Alpha*-diversity index of mannan polysaccharides and oligosaccharides at 24 h of fermentation.

Sample	Shannon	Simpson	Chao1	ACE
CK0	$5.16{\pm}0.06^{a}$	$0.95{\pm}0^{a}$	$303.27{\pm}19.43^{cd}$	$301.4{\pm}14.29^{c}$
CK24	$4.69{\pm}0.11^{a}$	$0.92{\pm}0^{a}$	$293.67{\pm}18.36^{cd}$	$293.39{\pm}22.12^{c}$
K1	$3.15{\pm}0.03^{cd}$	$0.74{\pm}0.01^{c}$	$189.75{\pm}4.73^{d}$	$196.39{\pm}5.03^{d}$
K2	$2.97{\pm}0.16^{d}$	$0.70{\pm}0.03^{c}$	$176.75{\pm}11.96^{d}$	$180.84{\pm}10.72^{d}$
L1	$3.61{\pm}0.2^{bc}$	$0.81{\pm}0.01^{b}$	$485.32{\pm}152.69^{abc}$	$493.47{\pm}158.84^{abc}$
L2	$3.78{\pm}0.07^{b}$	$0.82{\pm}0.01^{b}$	$611.66{\pm}22.69^{ab}$	$637.16{\pm}25.05^{ab}$
G1	$3.58{\pm}0.17^{bc}$	$0.80{\pm}0^{b}$	$362.17{\pm}155.4^{bc}$	$369.57{\pm}157.23^{bcd}$
G2	$4{\pm}0.22^{b}$	$0.82{\pm}0.01^{b}$	$686.13{\pm}41.47^{a}$	$695.7{\pm}47.23^{a}$

Note: CK0: blank control at 0 h fermentation; CK24: blank control at 24 h fermentation; K1: konjac polysaccharides; K2: konjac oligosaccharides; substrate K (K1 and K2); G1: guar polysaccharides; G2: guar oligosaccharides; substrate G (G1 and G2); L1: locust bean polysaccharides; L2: locust bean oligosaccharides; substrate L (L1 and L2); Different lowercase letters indicate significant difference between samples (*P<0.05*).

Figure S1. Molecular Structure characteristics and their corresponding degradation pattern of konjac gum, guar gum and locust bean gum.

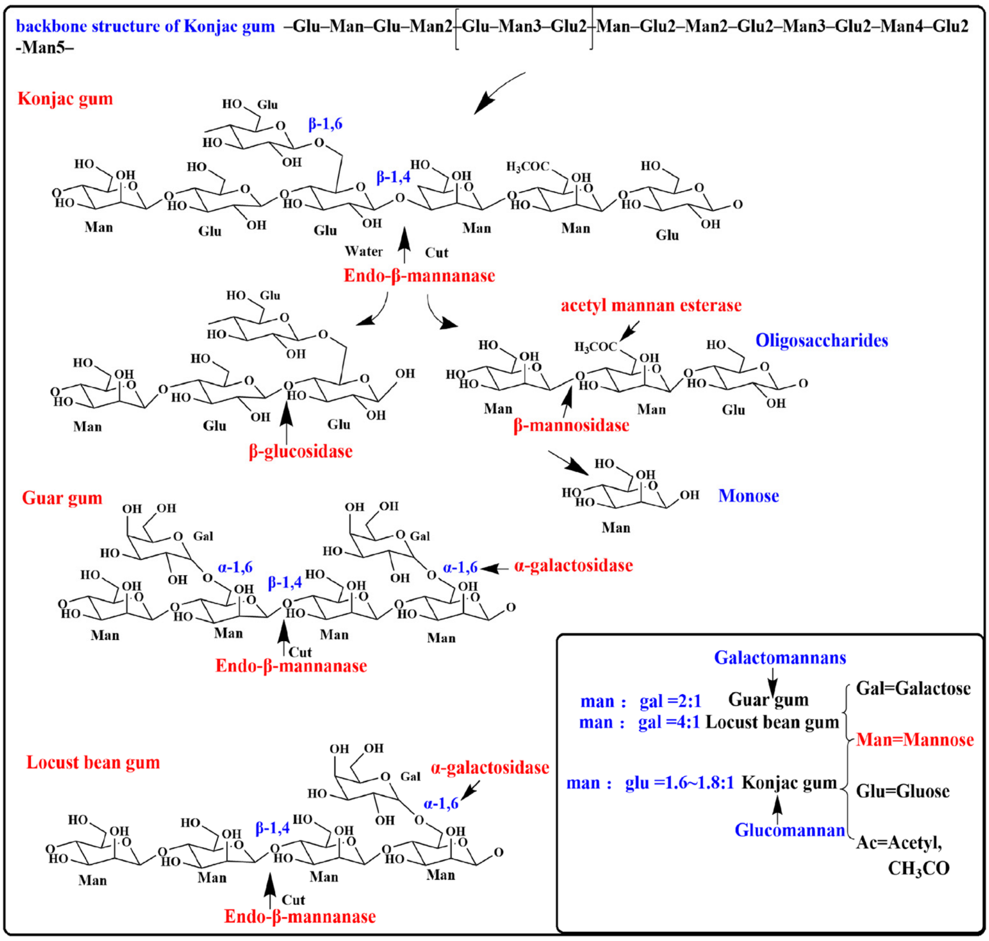

Supplement: Supplementary file 1 [file foods-12-04002-s001.zip › foods-2553968-supplementary.pdf]
